# Blockchain-Based Concept for Digital Transformation of Traceability Pyramid for Electrical Energy Measurement

**DOI:** 10.3390/s22239292

**Published:** 2022-11-29

**Authors:** Kruno Miličević, Ivan Tolić, Davor Vinko, Goran Horvat

**Affiliations:** 1 Random Red Ltd., 31000 Osijek, Croatia; 2 Croatian Transmission System Operator Ltd., 10000 Zagreb, Croatia; 3 Base58 Ltd., 31000 Osijek, Croatia

**Keywords:** digital transformation of metrology, blockchain, electrical energy meter, traceability pyramid, European Metrology Cloud, Metroracle project

## Abstract

Digital transformation of metrology is a holistic process that was started formally by the Joint Statement of Intent “On the digital transformation in the international scientific and quality infrastructure” signed by major metrology organisations in March 2022. With the digital transformation of metrology in motion, the questions of a seamless transition to digital representation while ensuring adherence to all the requirements of digital representation and maintaining a sustainable framework for future operations are just some of the challenges faced. To tackle these challenges, also within the concept of “more electrical world” (MEW), one technology is demonstrating high potential applicability as a possible candidate solution—blockchain technology, with its critical underlying properties (e.g., immutability, decentralisation, etc.) being fully compliant with the requirements of digital representation in metrology. Accordingly, this paper presents a blockchain-based concept for the digital transformation of the traceability pyramid for electrical energy measurement. The concept is developed in accordance with the goals of the Metroracle project. Based on the analyzed and presented state-of-the-art, the main contribution of the paper is the comprehensiveness of the concept, which encompasses the whole pyramid and describes all relevant processes and responsibilities of all stakeholders: measurement instrument (MI) owners, certificate issuers (National Accreditation Body (NAB), National laboratory (NL), Reference laboratory (RL)), MI manufacturers/developers, MI installers. The transformed pyramid is defined by Croatian metrology laws and regulations, but with smaller adjustments, it is applicable to other EU countries as well, and also to the traceability of other physical quantities, i.e., not to electrical energy only. Possible legal and technical issues are identified (amount of data, machine-readable standards and regulations, ensuring limited access, legal relevance of digital signature) and corresponding solutions presented, as well as further steps in our research and development within the Metroracle project.

## 1. Introduction

Electrical energy measurements are unavoidable components in the concept of a “more electric world” (MEW). One of the most important parameters of any energy conversion system is energy conversion system efficiency, which is defined as the ratio of useful energy output and energy input [1]. Hence, the main role of sensor of the electrical energy (SEE) and corresponding measuring instrument (MI) in the MEW is the monitoring of power flows with the aim of improving the performance of modern energy conversion systems, as well as monitoring smart grid systems [2,3,4]. An accurate ensemble of SEE and MI is of key importance for the operation of these systems, which is confirmed through the legal metrology framework.

Metrology, although a traditional discipline developed patiently through the decades (e.g., Meter Convention in 1875, International System of Units in 1960), nowadays faces the opportunities and challenges of information and communication technology, i.e., digital transformation. The critical property of a measurement result is traceability: any result can be related, through the finite chain of comparisons, to the primary definition of measurement quantity. This property is practically realized by establishing a measurement traceability pyramid (MTP), which represents a traceable chain of relationships from a measuring instrument (sensor) up to international measurement standards [5,6]. An MTP can be separated into two paths. The first is the path of the MI, from production, through the reference laboratory (RL) to the customer. The second path is the path of the reference standard, from a calibration performed by a national (primary or secondary) laboratory to the accreditation in its parent laboratory. The latest efforts on the international level are directed at the digital transformation of metrology through information technology.

This process is recognised and supported in the metrology community, as it is visible in the Joint Statement of Intent “On the digital transformation in the international scientific and quality infrastructure” signed by major metrology organisations: The International Bureau of Weights and Measures (BIPM), Committee on Data of the International Science Council (CODATA), International Organization for Standardization (ISO), International Organization of Legal Metrology (OIML), The International Measurement Confederation (IMEKO) [7]. In this manner, many international task groups are established [8,9] to encourage continuous development in this sense, e.g., by jointly organising thematic workshops [10,11].

The efforts of metrology organisations are followed by recent scientific papers dealing with challenges in the digital transformation of metrology [12]. Authors in [13] design a multi-layered modular digital framework to comprehensively digitalise metrological information. The possible application of blockchain technology, considering the entire MTP, is presented in our previous work [14].

Our concept is developed within the Metroracle project [15]. Metroracle project focuses on adopting well-defined metrology standards for use in IoT applications supported by blockchain technology. In this paper, the Metroracle approach is applied to electrical energy measuring, and metrology standards are contained in the MTP, which is the backbone of legal metrology. The concept includes the transformation of the whole MTP for electrical energy measurement, i.e., all relevant processes and responsibilities of all stakeholders: MI owners, certificate issuers (National Accreditation Body (NAB), National laboratory (NL), Reference laboratory (RL)), MI manufacturers/developers, MI installers.

In Section 2, we present a traditional MTP and its current properties.

The main goal of this paper is to define a strategy for migrating traditional MTP to its digital counterpart (Section 3). This process is of crucial interest for legal metrology information and any aspect of life where metrological data is used.

Aiming to clarify the theoretically described process of digitalisation of MTP, this paper in Section 4 presents a blockchain-based proposal for the digital transformation of the pyramid for an energy meter, through the calibration chain in Croatia, which is comparable to other EU states as well. The process that starts with the production of an electrical energy meter and ends at the customer’s facility, as well as associated documentation, will be described in detail, with an emphasis on the digital transformation of the process and its technical and legal challenges.

## 2. State of the Art—Traditional Measurement Traceability Pyramid

In this section, we present the state-of-the-art on MTP, which is to be translated into its digital counterpart. It is worth noting that, in this paper, we refer to the legislation related to the European Union (EU) market and the Croatian market as its member. For more details or updated information about EUR-Lex content, the interested reader is directed to the Official Journal of the European Union, which is published daily in all EU official languages [16].

### 2.1. International Quality Infrastructure

**Figure 1:**
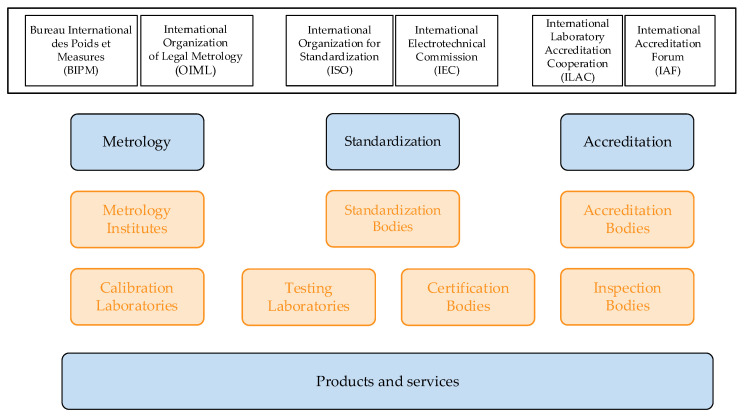
An OIML diagram for international quality infrastructure.

Any aspect of metrological activity is always related to quality infrastructure (QI). According to the OIML Bulletin [17], the basis of international QI includes metrology, standardisation, and accreditation (Figure 1).

Organisations responsible for the harmonisation of international QI are BIPM and OIML (metrology institutes—calibration and testing laboratories), ISO and IEC (standardisation bodies), and The International Laboratory Accreditation Cooperation (ILAC) and International Accreditation Forum (IAF) (accreditation bodies—certification and inspection bodies). Harmonisation in this aspect means successful cooperation between all stakeholders, e.g., from manufacturers and their associations, through regulatory bodies to previously mentioned international organisations participating in QI. A particular challenge in the digital transformation of metrology is standardisation. This part of the QI is challenging to transform in a machine-readable way due to the unambiguous legal interpretation of its provisions. Hence, this paper focuses on the metrology and accreditation aspects of QI, i.e., without the standardization aspect.

**Figure 2:**
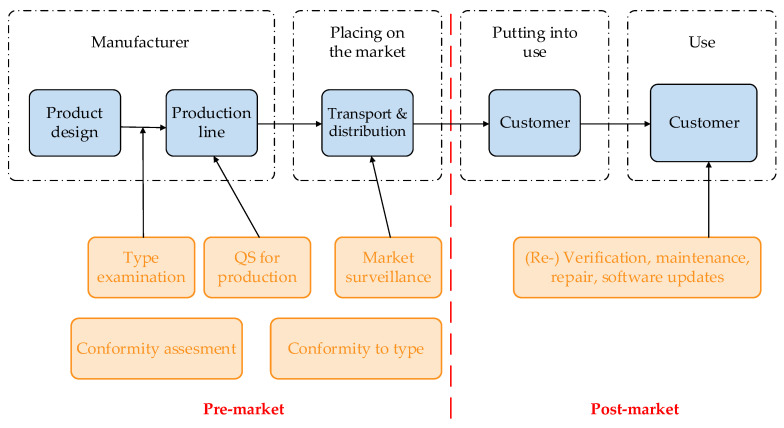
The life-cycle of the product separated into pre-market and post-market activities.

From the OIML point of view, the product, e.g., MI, life cycle could be separated into pre-market and post-market activities (Figure 2). Pre-market activities are related to all steps before the customer has bought the product (manufacturing, conformity assessment, EU-type approval, etc.). Post-market activities are connected to all actions after the customer has purchased the product (verification, maintenance, repair, etc.). OIML states that digital transformation, considering the whole lifecycle of the product, could be successfully performed only if it is based on the so-called FAIR+T principle: data that is Findable, Accessible, Interoperable, Reusable, and Traceable to the SI [14,17].

### 2.2. Path 1 of Measurement Traceability Pyramid

**Figure 3:**
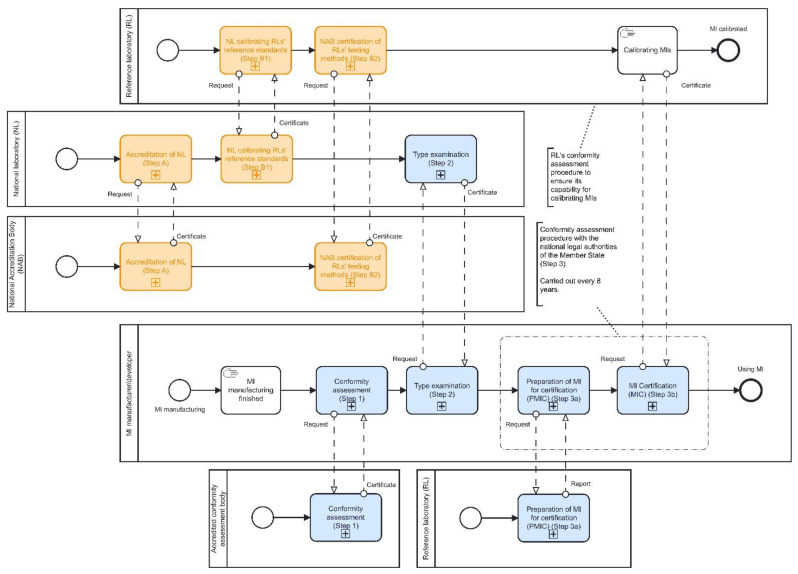
BPMN diagrams for the international hierarchy scheme of the reference standard (Path 1—blue coloured) and MI (Path 2—orange coloured).

As previously mentioned, MTP can be separated into two paths. Figure 3 shows corresponding BPMN diagrams that cover metrology and accreditation aspects (see Section 2.1). Thereby, the diagram mostly indicates the pre-market phase of the product life-cycle (see Figure 2). The post-market phase is related to steps “Using MI” and to Steps 3a and 3b due to the periodic nature of MI certification.

The first path (Path 1—marked blue in Figure 3) starts with the production process of MI. A manufacturer is obliged to ensure that the production process is aligned with Directive 2004/22/EC of the European Parliament and of the Council of 31 March 2004 [18], Regulation (EC) No 765/2008 of the European Parliament and of the Council of 9 July 2008 [19] and other EU harmonisation legislation which stem from the latter directive. To demonstrate that MI complies with the essential requirements of the EU market, a manufacturer should pass through conformity assessment procedures according to [20] (Step 1). Visible consequences of the conformity assessment procedure are CE marking and supplementary metrology markings.

In Step 2, MI is ready to be released to the market of the EU Member State. A necessary point at this moment is an EU-type examination according to [18]. It is part of the conformity assessment procedure where the notified body examines if the specified technical characteristics of the MI comply with the requirements of the EU market and issues the EU-type examination certificate. This certificate has a validity of 10 years from the date of its issuing. The National Metrology Institute (NMI) of the Member State could accept the latter certificate or perform its type of examination. In the example provided in this paper, the Croatian NMI can perform the examination type according to [21].

When MI is released to the market, before it is ready to use, it must pass the conformity assessment procedure with the national legal authorities of the Member State (Step 3). Croatia’s conformity assessment procedure is divided into two sub-steps [21]. The first step (Step 3a) is the preparation of MI for certification (PMIC), which is performed in the reference laboratory certified by the NMI. In this step, MI is tested in predefined testing points. If the test is passed successfully, an authorised person of the RL signs a Test Report. PMIC is a necessary step before Step 3b, MI Certification (MIC). The MIC process is performed in a reference laboratory certified by NMI and accredited by the National Accreditation Body (NAB) according to EN ISO/IEC 17020 standard. If MIC is certified successfully, an authorised person signs a verified Certificate of Calibration and applies seals which are physical protection from manipulation on MI. After the first iteration of PMIC and MIC processes in the life cycle of the MI is finished, it is ready to use. This signals the end of the pre-market phase. All future activities are related to the post-market phase.

PMIC and MIC processes are performed in subsequent periods of eight years according to the Croatian national legislation in the example of the energy meter. Steps 1–3 apply for every single energy meter passing through Path 1 of MTP (Figure 2).

### 2.3. Path 2 of Measurement Traceability Pyramid

Path 2 (marked orange in Figure 3) of MTP focuses on the reference standard. This path starts with Step A where the NAB issues a Certificate of Accreditation (CA) to the national (primary or secondary) laboratory (NL) according to EN ISO/IEC 17025. The NL can then calibrate reference standards used by RL with lower measurement capability. Step A is performed in subsequent periods of two years according to the Croatian national legislation.

In Step B, RL 2 passes through a conformity assessment procedure to ensure its capability for calibrating MIs. In the first part of this step (Step B1), NL or other RL 1 with higher measurement capability calibrates its reference standard, hence approving its technical competencies. In Step B2, NAB issues a Certificate of Accreditation to RL 2 and confirms its competence in performing accredited testing methods. In a particular example, RL achieves competencies for calibrating energy meters.

### 2.4. Associated Documentation

Table 1 presents an overview of documentation issued through the previously given steps. For each step, the table shows which document is signed, what it approves, and the associated validity period. It is worth noting that the validity period corresponds to the particular example of energy meter and reference standards covered by Croatian national legislation. It may generally differ for other types of MI and other types of reference standards.

### 2.5. A Comparison of Calibration Chains in Croatia, Germany, the United Kingdom and Estonia

Although national legal regulations are directed towards international quality infrastructure organised by NMIs and international organisations (such as EURAMET, WELMEC, CIPM, BIPM, ISO, etc.), these regulations are not fully harmonised. In this section, we will compare the metrology infrastructure of Croatia with three international examples: Germany, the United Kingdom, and Estonia. This comparison is focused on the calibration chain as a critical part of the international quality infrastructure [22].

Table 2 presents national hierarchy schemes in the compared countries. The first difference is at the national level where Croatia, Germany, and Estonia have a single national metrology body: CNMI, PTB and COM, respectively, while in the United Kingdom it is divided into standard (NPL) and legal (OPSS) metrology organizations. The second difference is at the level of RL1, where Croatia and Germany have private accredited bodies, while the United Kingdom and Estonia have national standard laboratories.

The third difference is at the level of RL2, where the United Kingdom has private accredited bodies. Still, the other three countries have other reference laboratories with lower measurement capabilities, e.g., in-house calibration laboratories. The last level is the same for all compared countries. It is customers´ level of use, e.g., measurements in production processes. Table 3 shows the names of NABs in compared countries and a comparison considering the accreditation status of NMIs and their capability of issuing Digital Certificates of Calibration (DCC).

The role of NMIs in the calibration chain is to issue a Certificate of Calibration to lower-level RLs. NMIs in Germany, the United Kingdom, and Estonia, unlike Croatian NMI, are accredited against EN ISO/IEC 17025, i.e., are capable of issuing a Certificate of Calibration. The next main difference between NMIs is the capability of issuing DCC. NMIs in Germany, the United Kingdom, and Estonia, unlike Croatian NMI, issue PDF versions of the Certificate of Calibration. None of the compared NMIs are capable of issuing machine-readable DCC at this moment. A significant effort on an international level, led by PTB, is directed at creating fully machine-readable DCC [23]. NABs in compared countries are harmonised under European co-operation for Accreditation (EA).

## 3. State of the Art—Digital Transformation of Metrology

In October 2020, a new BIPM/OIML Joint Task Group (JTG) was established to, among other goals, promote and support the digital transformation of metrology, e.g., the JTG:

- Recognise the necessity for a digital transformation of industrial, legal, and scientific metrology activities and processes in close cooperation with all stakeholders in the field of quality infrastructure;
- Support that the digital representations of physical devices should rely on robust, unambiguous, and machine-actionable data, using the SI and aiming at meeting the FAIR principles to facilitate efficient processes in industry, economy, society, modern research, and global development.

Consequently, the digital transformation of metrology requires a holistic approach for all parties working in quality infrastructure [17]. It is important to mention that there are many cryptographic techniques to secure digital transactions and data exchange; for example, asymmetric encryption, digital signatures and corresponding PKI, and their various applications, but due to the holistic nature of the digital transformation of metrology, it is needed to combine them into a platform used by all relevant stakeholders, i.e., a platform which will fulfil all requirements listed in Table 4. The most prominent and, to the best of the authors’ knowledge, the only holistic concept at the moment, is the European Metrology Cloud (EMC) developed by PTB, which is designed to support the processes of conformity assessment and market surveillance related to metrology [24]. Its main objectives are [25]:

- Objective 1: A trustworthy metrological core platform—implement digital concepts for the coordination, concentration, simplification, harmonization, and quality assurance of metrological services for the Member States and all parties involved;
- Objective 2: Reference architectures—provide and distribute knowledge via broadly applicable general reference architectures specifically for new and complex technologies fitting the needs of all stakeholders, i.e., the requirements established by the regulations;
- Objective 3: Technology-driven metrological support services—change processes related to European legislation coordinating the interaction of a large variety of partners in legal metrology in a way that would be most beneficial regarding the organization of market surveillance and verification, the services of notified bodies and manufacturers, as well as the needs of the users of measuring instruments;
- Objective 4: Data-driven metrological support services—develop data-based services for legal metrology, a theoretical approach taking advantage of the available data sources created by all stakeholders.

**Table 4:** Requirements for digital representations in metrology and blockchain as a possible solution [14].

Requirements for Digital Representations in Metrology		Blockchain Properties		Recommendations/Possible Issues
- must contain all the relevant information that the responsible bodies need to perform: conformity assessment, verification, and market surveillance in a machine-readable way		Programmable data structures with immutability aspect and access control		Issue 1—Volume of data
- must “know” the relevant standards and regulations, and provide machine-readable information about them				
- must contain all the relevant information for customers so that they may gain trust and confidence in the products and quality measures		Blockchain inherently uses machine-readable information		Issue 2—Bridging the gap between the blockchain standards and machine-readable standards/regulations in metrology
- must provide machine-readable interfaces for users and manufacturers to enable smart quality assurance				
- must combine machine-readable documents and certificates, hence enabling automation of the digital QI processes				
- must be secured and validated to provide access to information only to eligible parties (for example by the use of blockchain technology)		Blockchain uses asymmetric cryptography to validate actions and changes in data		Issue 3—Ensuring limited access in an inherently public technology Issue 4—Legal relevance of blockchain-based digital signature

Although it is holistic (or exactly because of the holistic approach), one of the key EMC’s challenges is the investigation of the potential of combined data from different sources, i.e., establishing a metrological data lake. One of the data sources for the lake could be blockchain-based data recordings, and this possible role of Metroracle is already actively discussed in collaboration with PTB’s Department “Metrology for the Digital Transformation”.

**Figure 4:**
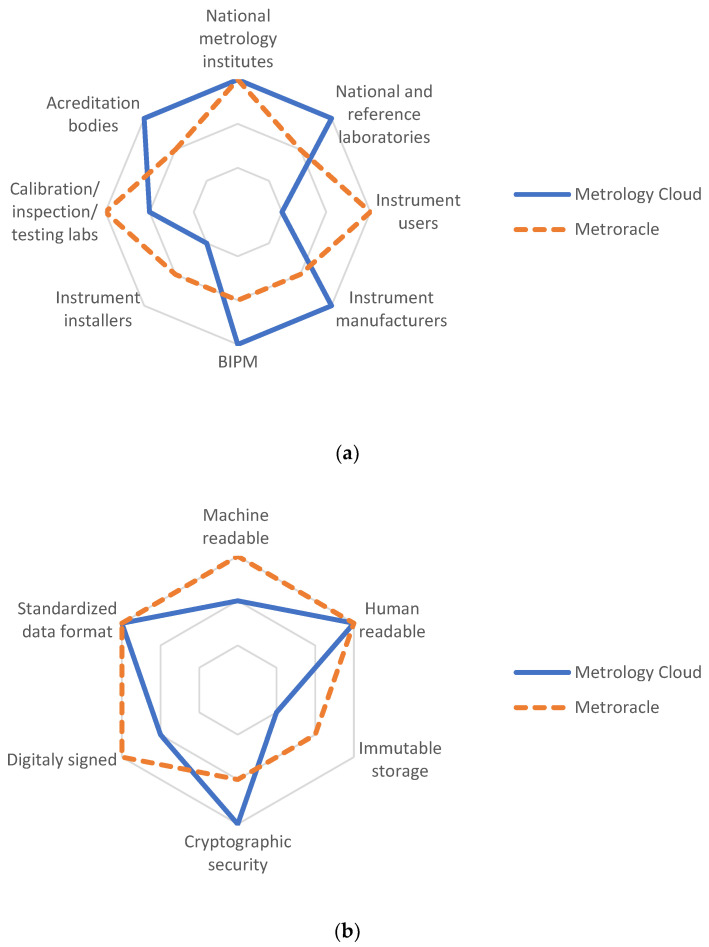
EMC and Metroracle scope comparison for (**a**) stakeholders and (**b**) features.

To illustrate the scope of both EMC and Metroracle, Figure 4 shows to which extent are the stakeholders and features “digitized”. There are three level of digitalization that are shown in Figure 4. The lowest level (e.g., Instrument users and Instrument installers for Metrology Cloud on Figure 4a) corresponds to either classical paper-based metrology, which is not digitized or to non-supported stakeholder or features. The second level (e.g., Immutable storage and Cryptographic security for Metroracle on Figure 4b) corresponds to stakeholders and features that are either partially supported or optional, i.e., they are not the primary stakeholders/features. The highest level (e.g., Acreditaion bodies and National metrology institutes for Metrology Cloud in Figure 4a) corresponds to a fully supported stakeholder/feature. It can be seen that EMC and Metroracle are complementary solutions whose synergy present a valuable addition to the digital transformation of metrology.

### 3.1. Blockchain as Metrology Infrastructure

In addition to the role of a pure database, the blockchain satisfies some additional requirements of legal metrology [17] (Table 4). However, blockchain technology does exhibit various challenges, which are discussed in more detail at the end of this section.

There are possible blockchain applications in various areas [26,27,28,29]. At the moment, several blockchain use cases are related to the traceability pyramid and legal metrology [30]:

- collaborative blockchain network for NMIs [31]
- distributed measuring systems where legally relevant (LR) software [32] are implemented on a blockchain [33,34]
- public-key Infrastructure for Smart Meters using Blockchains [35]
- field surveillance of measuring instruments [36]
- all of these could be implemented in our concept of a digitalized traceability pyramid due to its holistic nature.

On the other side, blockchain is also proposed as a technology applicable also to electrical energy metering [26]. However, the emphasis is mostly on billing [37,38]. Our concept does not consider the billing procedure, but of course, the implementation of a digitalized traceability pyramid is the basis for advanced billing systems as well.

It is common to assume high computational complexity and energy demand for blockchain. However, this is an issue inherent to the “proof-of-work” consensus used, e.g., for Bitcoin Blockchain. In our case we would avoid this kind of approach; i.e., some other kind of consensus type could be used, like “proof-of-authority” or “proof-of-stake”, as explained in more detail in [14].

#### 3.1.1. Issue 1—Amount of Data

The distributed nature of blockchain technology dictates that data is stored in a decentralised manner (present in all nodes supporting the blockchain network). Increasing numbers of MIs that require certification would, in time, lead to an increase in the volume of data stored on the blockchain, resulting in the need for increasing data storage capacities (blockchain bloat). Accordingly, large volumes of data should not be stored within the scope of blockchain technology. This problem is not isolated to blockchain usage in metrology, but rather a common technological limitation of blockchain technology, when used for data storage in general.

The effects of data bloat can be mitigated by storing the data off-chain (i.e., on a server outside of the blockchain infrastructure) and the digest of the data on the blockchain. This would ensure the immutability of the off-chain stored data while balancing the data growth issue on the blockchain. General solutions for data growth in blockchains include various approaches, one of them being sharding blockchain systems [39].

#### 3.1.2. Issue 2—Machine-Readable Standards and Regulations

To avoid an overload of information and details provided to end-customers, one should provide customers with the minimum of information, e.g., binary information on the regulatory fulfilment of certain standards. This can be achieved by smart contract concepts within blockchain technology that are executed automatically when the defined conditions are fulfilled [40,41].

Thus, the formats of machine-readable documents and certificates must have a clear logical structure, to avoid any misunderstanding and misinterpretation of data and, consequently invalid execution of smart contracts.

To ensure interoperability, the structure format should also be widely accepted by the metrology community, which is already a topic, for example in the case of digital calibration certificate (DCC) [42,43].

#### 3.1.3. Issue 3–Ensuring Limited Access

To ensure controlled access to the information, it is necessary to select a corresponding blockchain type based on the CIA (Confidentiality, Availability, Integrity) assessment. Namely, in a public blockchain infrastructure, all transactions are publicly visible and it is possible to add transactions for every user, without any limitations. However, in metrology applications, access should be possible only to eligible parties. In such cases, it is recommended to use a blockchain administered by one entity (private) or by more entities (consortium blockchain), where the latter seems to be a more appropriate approach due to the more than one level of responsibility in the metrology hierarchy, e.g., consortium could be established by NMIs, NABs, RLs, and NLs.

#### 3.1.4. Issue 4—Legal Relevance of Digital Signature

Digital signatures are used for authenticating users and devices in the digital world. The technology is based on asymmetric encryption, where a particular cryptographic key pair belongs to a particular user:

- private key securely stored, i.e., accessible only to the key owner
- public part of the key exposed for public use

It ensures:

- Confidentiality: No entity can view a payload in clear text without owning the private key.
- Integrity: The content of transmitted data is signed with a private key and then verified using the public key.
- Authenticity: Certainty of what you are connecting to, or evidencing your legitimacy when connecting to a protected service.

However, in legal metrology, (legal or physical) persons, as shown in Table 1, sign various documents within the metrology hierarchy. In order to make these signatures legally valid in digital form, it is necessary to link the public key with the identity of a person. Thereby, this link must be guaranteed by a “trusted third party” (e.g., Trust Service Providers, TSP), and this procedure is defined in eIDAS regulation [44], which defines a Qualified electronic signature as the one legally equivalent to a handwritten signature.

Even though blockchain technology is explicitly mentioned in [17,45] as a possible solution for secure and validated access to information only to eligible parties, it has the disadvantage of not being regulated under eIDAS in terms of digital signature (transactions signed are not a part of PKI infrastructure). In turn, this presents an issue when attempting to confirm the identity of the signer automatically via digital signatures.

It is worth noting that this problem is not isolated to legal metrology, but rather a general problem of legal relevance related to blockchain signatures. Fortunately, this problem is already recognised by legislator bodies in the EU and the solution is planned in the form of the eIDAS SSI bridge [46]. This solution would be applicable in legal metrology as well.

## 4. Digital Transformation of the Pyramid for Energy Meter

Section 4 presents a practical example of the digital transformation of the pyramid for energy meters, through the calibration chain in Croatia, which is comparable to other EU states as well. For this use case, we can identify the following main stakeholders and their activities, which start with the production of an electrical energy meter and end at the customer´s facility:

- MI owner—Install MI; call MI installers for a higher trust level (optional); connect MI to blockchain and claim ownership; administrate MI options; define the frequency of data measurements; define the batch size of data measurements; view MI status and properties (device activity, ownership, appended trust indicators, e.g., various certificates, anti-tampering alarms, etc.); make recorded measured data accessible (to limited user types or completely public)
- certificate issuer (National Accreditation Body (NAB), National laboratory (NL), Reference laboratory (RL), see Figure 3)—Certificate/test MI; issue certificates according to legal metrology or raise trust even without legal obligation and record them on a blockchain infrastructure.
- MI manufacturer/developer—Manufacture MI; connect MI to blockchain infrastructure and claim (initial) ownership, i.e., before first selling
- MI installers—Install MI; issue certificates on installing the device and record them on a blockchain infrastructure.

**Figure 5:**
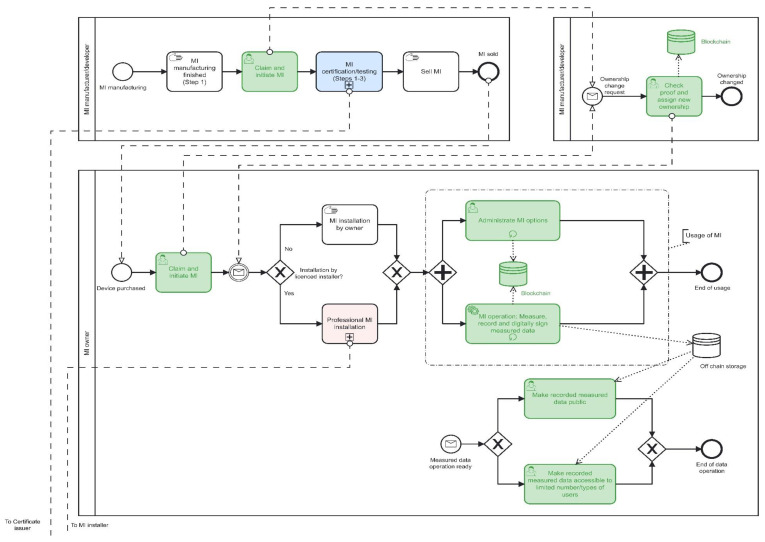
A BPMN diagram showing processes related to MI manufacturer/developer and MI owner.

**Figure 6:**
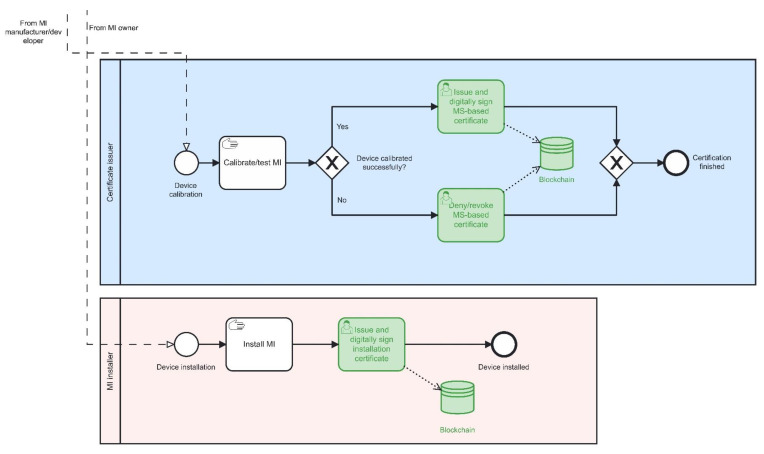
A BPMN diagram showing processes related to certificate issuers and MI installers.

Activities are presented as BPMN diagrams shown in Figure 5 and Figure 6. Thereby:

- green-coloured blocks present elements of digital transformation, i.e., activities recorded on a blockchain infrastructure (or in off-chain storage, due to Issue 3.1);
- step “MI certification/testing (Steps 1–3)” corresponds to all those steps shown in Figure 3, i.e., the digital procedures shown in Figure 5 and Figure 6 are in the same way applicable to Steps 1–3 (Path 2) demonstrated in Figure 3. As a result, each issued certificate (see Figure 3) must be digitally signed by the corresponding authority (see Table 1 and Issue 3.4);
- Steps A and B (Path 1) are not comprised in Figure 5 and Figure 6. Still, they could be digitally transformed in the same way; e.g., each certificate request and issuing (including digital signature) could be recorded on a blockchain infrastructure.

There are added functionalities:

- digital recordings about (initial) claiming and selling the device, i.e., changing the ownership;
- administrating MI options, e.g., define the frequency of data measurements, define the batch size of data measurements, view device status and basic properties (device activity, ownership, appended trust indicators, e.g., various certificates, anti-tampering alarms, etc.);
- making recorded measured data public or accessible to a limited number/types of users.

## 5. Conclusions

The digital transformation of metrology is a holistic process and a huge challenge for the whole metrological community, from the top of the metrology pyramid down to the bottom, i.e., end-users of measuring instruments and measurement results.

In order to preserve trust in measurement results and traceability, built through decades of metrology development, it is necessary to approach the digital transformation with the highest caution. The paper shows the concept of digital transformation of the metrology pyramid for an energy meter, through the calibration chain in Croatia, which is comparable to other EU states as well. The concept is based on blockchain infrastructure, utilising properties such as immutability and cryptographic security.

Based on the analyzed and presented state-of-the-art, the paper’s main contribution is the concept’s comprehensiveness—it encompasses the whole pyramid for the electrical energy measurement, describing all relevant processes and responsibilities of all relevant stakeholders.

Although technically feasible, the concept reveals several issues, such as a limited amount of data stored in the blockchain, machine readability of standards and regulations, regulated access of blockchain data, and legal relevance of digital signatures. These issues are not peculiar to metrology only, and corresponding solutions, such as eIDAS SSI Bridge for digital signatures, have already developed in the blockchain community. Thus, in the future development of the proposed concept, through the Metroracle project, we will address the application of those solutions to the metrology traceability pyramid, and further development and adjustments of our concept according to the needs of all metrology stakeholders (national metrology institutes, national and reference laboratories, and users of measuring instruments).

Furthermore, in correspondence with the concept, we will build the blockchain platform, needed interfaces for stakeholders, and a measuring device prototype to record the measuring data to the blockchain and append to them certificates issued by calibration laboratories. The results of implementation will be validated, including the qualitative and quantitative analysis. The success of the presented concept and Metroracle project, in general, depends heavily on the participation of the metrology community in providing relevant metrology data. Thus, this paper also aims to invite the metrology community to participate in our further research and to test developed solutions, such as were already discussed with PTB’s Department “Metrology for the Digital Transformation” related to the synergy with their European Metrology Cloud project.

## Figures and Tables

**Table 1:** An overview of documentation issued through the steps of MTP.

	What Is Signed?	What Is Approved?	Period of Validity	Who Signs?
Step 1	Certificate of Conformity Declaration of Conformity	Production process Product	3 years	CEO on behalf of the institution
Step 2	Type examination certificate	Type of MI	10 years	CEO on behalf of notified body
Step 3A	Test Report	Particular MI	8 years	Authorized person on behalf of RL
Step 3B	Verified Calibration Certificate	Particular MI	8 years	Authorized person on behalf of RL
Step A	Certificate of Accreditation	Accredited testing methods	2 years	CEO on behalf of NAB
Step B1	Calibration Certificate	Particular reference standard	2 years	Authorized person on behalf of NL or RL 1
Step B2	Certificate of Accreditation	Accredited testing methods	2 years	CEO on behalf of NAB

**Table 2:** National hierarchy schemes of calibration chains in Croatia, Germany, the United Kingdom, and Estonia.

	Croatia	Germany	United Kingdom	Estonia
**National** **Laboratory**	Croatian National Metrology Institute (CNMI)	Physikalisch–Technische–Bundesanstalt (PTB)	National Physical Laboratory (NPL)	The Central Office of Metrology (COM)
			Office for Product Safety and Standards (OPSS)	
**Reference** **Laboratory 1**	Accredited Calibration Laboratories	Accredited Calibration Laboratories	Designated National Laboratories such as National Engineering Laboratory (NEL), National Measurement Laboratory (NML), etc.	National standard laboratory AS Metrosert
**Reference** **Laboratory 2**	Other Reference Laboratories	Other Reference Laboratories	Accredited Calibration Laboratories	Other Reference Laboratories
**Customers**	Industry	Industry	Industry	Industry

**Table 3:** A comparison between NMIs’ role in Croatia, Germany, the United Kingdom, and Estonia.

	Croatia	Germany	United Kingdom	Estonia
**National Metrology Laboratory**	Croatian National Metrology Institute (CNMI)	Physikalisch-Technische Bundesanstalt (PTB)	National Physical Laboratory (NPL)	The Central Office of Metrology
			Office for Product Safety and Standards (OPSS)	
**National Accreditation Body**	Croatian Accreditation Agency (CAA)	Deutsche Akkreditierungsstelle GmbH (DAkkS)	The United Kingdom Accreditation Service (UKAS)	Estonian Accreditation Centre (EAK)
**Accreditation against EN ISO/IEC 17025**	No	Yes	Yes	Yes
**Issues signed PDF version of Certificate of Calibration**	No	Yes	Yes	Yes
**Issues machine-readable DCC**	No	No	No	No
